# A comparative study of small-scale fishery supply chains’ vulnerability and resilience to COVID-19

**DOI:** 10.1007/s40152-021-00231-4

**Published:** 2022-01-01

**Authors:** Hannah R. Bassett, Sonia Sharan, Sharon K. Suri, Sahir Advani, Christopher Giordano

**Affiliations:** 1 School of Aquatic and Fishery Sciences, University of Washington, Seattle, USA; 2 Oceana, Washington, D.C USA; 3 Department of Anthropology and Department of Geography, Planning and International Development Studies, Amsterdam Institute for Social Science Research, University of Amsterdam, Amsterdam, The Netherlands; 4 Dakshin Foundation, Bengaluru, India; 5 Future of Fish, Lima, Peru

**Keywords:** Small-scale fishery, Supply chain disruption, Resilience, Vulnerability, COVID-19, Adaptive capacity

## Abstract

The COVID-19 pandemic and response has significantly disrupted fishery supply chains, creating shortages of essential foods and constraining livelihoods globally. Small-scale fisheries (SSFs) are responding to the pandemic in a variety of ways. Together, disruptions from and responses to COVID-19 illuminate existing vulnerabilities in the fish distribution paradigm and possible means of reducing system and actor sensitivity and exposure and increasing adaptive capacity. Integrating concepts from literature on supply chain disruptions, social-ecological systems, human wellbeing, vulnerability, and SSFs, we synthesize preliminary lessons from six case studies from Indonesia, the Philippines, Peru, Canada, and the United States. The SSF supply chains examined employ different distribution strategies and operate in different geographic, political, social, economic, and cultural contexts. Specifically, we ask (a) how resilient have different SSF supply chains been to COVID-19 impacts; (b) what do these initial outcomes indicate about the role of distribution strategies in determining the vulnerability of SSF supply chains to macroeconomic shocks; and (c) what key factors have shaped this vulnerability? Based on our findings, systemic changes that may reduce SSF vulnerability to future macroeconomic shocks include: diversification of distribution strategies, livelihoods, and products; development of local and domestic markets and distribution channels; reduced reliance on international markets; establishment of effective communication channels; and preparation for providing aid to directly assist supply chains and support consumer purchasing power.

## Introduction

Small-scale fisheries (SSFs) are important contributors to the global seafood system and crucial components of food and nutrition systems. Nearly half of the world’s seafood, the most traded food commodity (Asche et al. 2015), is produced by SSFs (World Bank 2012) and has the potential to address global undernourishment and food security issues (Golden et al. 2016; Thilsted et al. 2016; Hicks et al. 2019). However, COVID-19 and associated mitigation measures are disrupting global supply chain functions and the roles of supply chain actors. In this study we consider the impacts of COVID-19 disruptions on SSF supply chain functions and the responses of actors through comparison of case studies in Indonesia, the Philippines, Peru, Canada, and the United States.

While global seafood trade is economically valuable, generating USD 277 billion in 2016 (FAO 2018), existing approaches to understanding shocks and disruptions in seafood supply chains overlook important, and increasingly recognized, wellbeing considerations of the estimated 158 million people in production and distribution (World Bank 2012; HLPE 2017). Seafood supply chains can be subjected to a variety of shocks or disruptions impacting production or distribution (Gephart et al. 2017); however, the field of supply chain disruption studies focuses predominantly on management of disruptions to continue provisioning (Xu et al. 2020), excluding additional social considerations. Beyond consumers and producers, global seafood trade includes market intermediaries and processors relying on SSF supply chain functions (Fabinyi et al. 2018; Stoll et al. 2018).

Globalization has increased the interconnectedness between SSFs and expansive international commodity chains, allowing access to new and distant seafood markets, while also exposing SSFs to new risks (Crona et al. 2016a; b). With risks no longer geographically bound, they can be transferred from global or regional scales to local actors via “ripple effects” (Ivanov et al. 2014) or “multiplier effects” (Abeysinghe and Forbes 2005), essentially a chain of impacts creating teleconnected vulnerabilities (Adger et al. 2009; Stoll et al. 2018). Shifts to global markets have also led to diversion of seafood from local communities, unsustainable fishing, changes to traditional SSF dynamics, and competition of inexpensive imports with locally produced seafood (Crona et al. 2016a). This context underlines the importance of understanding SSF supply chain actor vulnerability to macroeconomic shocks, such as COVID-19.

In this context, SSFs and actors within these social-ecological systems (SES) have disproportionately felt the impacts caused by COVID-19 (Bennett et al. 2020). With the pandemic’s origin in China and ensuing closure of their trade markets, disruptions have rippled outwards through connected seafood systems, affecting multiple locations and nodes of seafood supply chains. This “ripple effect” is illuminating the global food system’s vulnerabilities and power imbalances (HLPE 2020; Love et al. 2021) that may be further exacerbated by future macroeconomic shocks caused by climate change (Lam et al. 2020). As the pandemic continues, case studies are needed to document impacts on SSF supply chains (Bennett et al. 2020; Love et al. 2021) and highlight trends and mitigation or adaptation options. Identifying spatial disparity of impacts requires regional case studies (Meyer et al. 2020).

Examining SSF supply chain disruptions and responses requires integrating supply chain disruption, food systems, SES, and SSF systems theories and approaches. The supply chain disruption literature provides valuable conceptualizations of supply chain operations, disruption models, and phases of recovery (e.g., Ivanov et al. 2014; Golan et al. 2020; Xu et al. 2020). However, it narrowly defines supply chain resilience as continued provision of food products to consumers in support of food security (e.g., Tendall et al. 2015). This definition is limiting, both in its normative conceptualization of resilience as inherently desirable, and its exclusion of wellbeing provisions to supply chain actors in its identification of seafood supply chains’ core functions. By drawing from the SES literature, system sustainability can be defined, in a broadly applicable sense, as the continued provision of *inclusive* wellbeing to system actors (Matson et al. 2016). A system’s core functions would include the various ways wellbeing is distributed amongst actors. Thus, when integrated with a definition of resilience as the extent to which a system can absorb change while still performing its core functions (Walker and Salt 2012), SSF supply chain resilience encompasses the extent to which a system has been able to sustain existing benefit distribution to all involved actors when exposed to a given hazard or disruption. This conceptualization rooted in SES theory considers that SSF supply chains’ core functions include not only provision of nutrients to consumers, but also provision of livelihoods and numerous dimensions of wellbeing to actors at every node of SSF supply chains.

We operationalize Adger's (2006) conceptualization of resilience, defined as “the magnitude of disturbance that can be absorbed before a system changes to a radically different state,” to assess the resilience of SSF supply chains to COVID-19 by examining impacts of and responses to the disruption; we then consider underlying causal mechanisms of change via analysis of system and actor vulnerability. As applied to SSF supply chains, we consider a “radically different state” to be one in which there is a substantial change to the primary type or volume of products traded, the process by which trade occurs, or the groups of actors involved in and benefitting from the supply chain system. Vulnerability is the combination of a system and its actors’ exposure and sensitivity to disruptions and their capacity to adapt (McCarthy et al. 2001). To characterize exposure, we consider the stressors’ magnitude, frequency, duration, and areal extent (Burton et al. 1993) in relation to each system and actor group. Sensitivity, considered to be the extent to which each system and actor group is affected by the disruption (Adger 2006), is represented for actors by their degree of dependence on the disrupted system for obtaining wellbeing benefits and for systems by their degree of dependence on the disrupted parts of the system for continued operation. Adaptive capacity is the ability of a system or actors to change and adjust to disruptions (ibid) and encompasses learning, flexibility, assets, organization, and agency (Cinner et al. 2018).

We employ a non-normative concept of resilience as a quality of the system that is neither inherently positive nor negative, with resilience as a desired system attribute when the system is in a preferred state, and undesirable when a system is in an unwanted state (Walker and Salt 2012). The “desirability” of a system would then be determined by its degree of sustainability, where a sustainable system is one that continuously provides inclusive and equitable wellbeing to all system actors (Matson et al. 2016). Vulnerability, defined as the susceptibility to being harmed, is inherently negative in nature, as is widely accepted in the SES literature (e.g., Adger 2006). Assessing the sustainability of SSF supply chains before and during the pandemic is outside the scope of this paper; however, this would be a logical next step. Understanding system resilience and sustainability separately is important, as a system may be resilient but unsustainable, or vice versa.

In this study, we ask (a) how resilient have different SSF supply chains and actors been to COVID-19 impacts; (b) what do these initial outcomes indicate about the role of distribution strategies in determining the vulnerability of SSF supply chains to macroeconomic shocks; and (c) what underlying conditions have shaped this vulnerability? This study builds on an examination of initial responses of SSF supply chains to COVID-19 published by Bassett et al. (2021), which includes four of the case studies addressed here as well as two others.

## Methods

In light of COVID-19-related limitations on in-person fieldwork, we adopted a multi-step methodology incorporating ongoing case study research complemented by virtual key informant interviews. Cases were selected based on authors’ pre-existing knowledge of and interactions with SSFs and their supply chains in five countries — Indonesia, the Philippines, Peru, Canada, and the United States. Our focus on these cases studies and applied methods served to minimize burdens on actors, build on existing connections, and cover a diversity of geographies, sociopolitical contexts, and distribution strategies.

### Data collection

Authors’ pre-existing knowledge of their respective cases established baseline understandings of each system prior to COVID-19. Information about each study site and SSF supply chain’s experience of and response to the pandemic was gathered from media and governmental reports, websites, newsletters, and semi-structured interviews with key informants. Effort was made to interview actors from several nodes in the supply chains.

Interviews with key informants aimed to improve the authors’ understanding of events that transpired since the onset of COVID-19 through September 2020. A collaboratively developed set of questions exploring SSF supply chain vulnerability and resilience were adapted by case due to logistical constraints, cultural and linguistic norms, and knowledge gaps. By taking these steps, we aimed to address limitations to conducting timely research and avoid unnecessary burden to actors in an already stressful period. Additional details concerning data collection for each case are included in Table 1.

**Table 1:** Case-specific data collection details

Processor Rio (LKR) and mobile traders (LKM), Langkat, North Sumatra, Indonesia (LK)	Data were collected during six months of ethnographic fieldwork in the province of North Sumatra, through participant observation and interviews with midchain actors. This was followed by five months of remote data collection focused on Langkat, comprising five interviews in conjunction with a local research assistant and ongoing discussions with key informants via WhatsApp.
Tañon Strait, Visayan Seas small-scale fisheries, Philippines (PH)	Information collected was supplemented by five key informants, with unique knowledge and personal experiences on COVID-19 impacts to the SSF, located in Cebu and Negros Island in Tañon Strait and the Visayan Sea. Informants provided information via phone calls, emails, and through social media communications.
La Tortuga and La Islilla high seas artisanal fleet, Peru (PE)	Data collection was part of an ongoing project to incorporate social and economic incentives in the Peruvian mahi Fishery Improvement Project (FIP). Informants were from the supply chain of the communities of Las Islilla and La Tortuga, in Paita province of northern Peru. In July and August 2020, six informal interviews over video calls were conducted with FIP participants.
Skipper Otto’s, British Columbia, Canada (SO)	One of the co-authors (author 4) worked part-time for SO’s distribution team between 2015 and 2019 and continues to be a member of the fishery. Key informant interviews with two SO staff members were complemented with analysis of member newsletters, blog posts, podcasts, and personal observations of seafood pickups at their Vancouver facilities.
Red sea urchin dive fishery, California, U.S. (RSU)	Prior to COVID-19, RSU fishery information was gathered via informal, exploratory interviews. After the onset of COVID-19, fishery and supply chain information was obtained during five telephone interviews with three key informants between March and August. These informants play roles in urchin diving, selling, and processing, as well as participate in the CSUC.

### Analysis

Case narratives were written by each respective author, then qualitatively assessed to address the questions shown in Table 2. Authors then identified similarities and differences in resilience and vulnerability and their potential causes across and between actor groups within their case. All cases were then reviewed and compared by the other members of the research team. This exploratory approach precluded collection of strictly comparable data, but importantly, allowed for key insights to emerge from the actors’ lived realities.

**Table 2:** Key theoretical framework terms and overarching questions guiding qualitative analysis

Theoretical framework term	Guiding questions	Sources
Resilience	Has the supply chain changed to a radically different state? Has the supply chain continued to perform its core functions as prior? Has there been a substantial change to the primary type or volume of products traded, the process by which trade occurs, or groups of actors involved in and benefitting from the system?	Adger 2006; Walker and Salt 2012
Vulnerability	What combination of a system or group’s exposure and sensitivity to disruptions and the capacity to adapt is seen?	Adger 2006
Exposure	To what degree has the supply chain and its actors (e.g., nodes or connections) been *exposed* to a stressor, i.e., experienced COVID-19-derived environmental or socio-political stress? Characteristics of stressors to consider are: magnitude, frequency, duration, and areal extent of the hazard.	Burton et al 1993
Sensitivity	To what degree is the supply chain and its actors *sensitive* to the stressor, i.e., affected by the environmental or socio-political stress?	Adger 2006
Adaptive capacity	What degree of *adaptive capacity* has the supply chain and its actors exhibited to COVID-19 disturbances? Domains of adaptive capacity to consider are: learning, flexibility, assets, organization, and agency.	Adger 2006; Cinner et al. 2018
Sustainability^1^	To what degree has the supply chain provided equitable and inclusive benefits to supply chain actors pre- and during COVID-19?	Matson et al. 2016

^1^Sustainability was not assessed in the analysis, however, was a consideration that shaped our theoretical framing and thinking

## Case studies

**Fig. 1:**
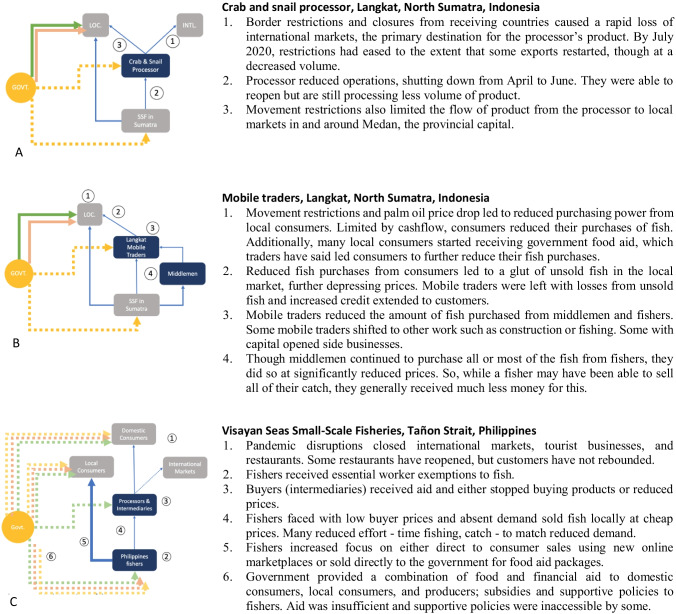

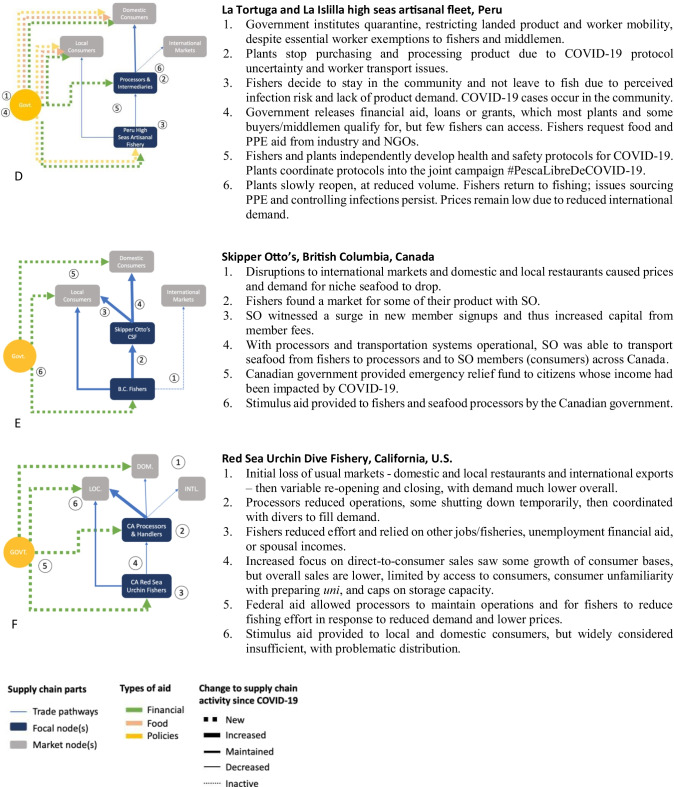
Diagrammatic representation of changes to SSF supply chains since onset of COVID-19, as of August, 31, 2020. Supply chain nodes are shown in rectangles, with focal nodes in dark blue and other nodes in gray. Supply chain pathways are shown as blue arrows and aid pathways are green, pink, and yellow, where line width and style reflect changes to trade and aid activity along the specific pathway. Numbers reflect the chronological and causal series of events and correspond to numbered descriptions at the right of each figure

Each case is described in terms of pre-pandemic operations and background and changes since the pandemic onset. Key events since the start of COVID-19 and intra-supply chain ripple effects are shown for each case in Fig. 1. The cases are presented in a way that allows for comparison, despite differing in scale and depth of detail around supply chain nodes, and with acknowledgement of the constraints of conducting research remotely during a pandemic.

### Processor and mobile traders, Langkat, North Sumatra, Indonesia

#### Pre-pandemic

Indonesia is the world’s second largest capture fish–producing country and a major exporter, with the North Sumatra province contributing 11% of volume and 12% of the associated monetary value (BPS-Statistics Indonesia 2020; FAO 2020). Indonesia has the highest nutritional dependence on marine and coastal ecosystems globally, with fish providing more than 50% of animal protein intake nationwide (Selig et al. 2019; FAO 2020). Within North Sumatra, the coastal regency of Langkat supplies a variety of supply chains with different endpoints, including local consumers, domestic consumers in the regional capital of Medan, and export markets such as Malaysia, China, Europe, and the United States (BPS-Statistics Indonesia 2020). Processors often work with only a few species, such as crab or shrimp, while mobile traders sell a wide assortment of fish from the local market, including tilapia, rays, mackerel, goatfish, and small shrimp, in addition to a mix of vegetables.

#### Since pandemic onset

By early February 2020, Indonesian seafood exports were being refused entry into China because of COVID-19-related restrictions, and customers in Langkat were wary of buying food imported from China (Fig. 1A1; LK01; LK02). Large-scale movement restrictions, *Pembatasan Sosial Berskala Besa*r, were implemented in early April through late May (CNN Indonesia 2020; Sianturi 2020). Although fishers and fish traders could continue working within Langkat, movement outside of the regency was restricted, limiting the movement of fish to Medan (Fig. 1A3). As the demand and price for palm oil, a major sector, declined sharply, consumers employed in the palm oil industry were laid off or saw their hours substantially reduced (LK3; Business Insider 2020).

Movement restrictions contributed to a seafood glut filling local markets, compounded by the reduced purchasing power of consumers from the palm oil sector (Fig. 1B1). More seafood in local markets with reduced consumer demand led to a sharp drop in seafood prices (Fig. 1B2). During a particularly low period in early May, two commonly consumed and affordable local fish, *Tongkol* and *Cuking*, dropped to 20% and 43% of their regular prices, respectively (pers. obs., S. Suri).

One crab and snail processor, Rio,^1^ noticed changes to the market from COVID-19 in late February, waiting until late March to close his factory. Exporters were the first to stop buying from him, then 3 weeks later, traders supplying the Medan market. Between pandemic onset and closing his factory, Rio limited his purchases to low-price products. After shuttering his business, he returned to working as a driver, but only for 1 month because of low demand. In July, some exports resumed, though volumes were far less than in previous years (Fig. 1A2; LK01).

Unlike the processors, mobile traders within Langkat continued to work throughout the pandemic; many households still relied on these traders for access to vegetables, tofu, tempeh, and fish. However, many customers were unable to work or took home reduced earnings, particularly those dependent on the palm oil industry (LK02, LK03). Some families received two or more months of government-distributed food aid, including rice, eggs, and oil, and/or small amounts of financial aid; small portions of dried anchovies used as seasoning were the only fish included in the food aid (LK03, LK04). Despite reduced fish prices and supplemental financial aid, local households reduced their fish purchases, with food aid potentially displacing demand.

Local consumers continued to buy from mobile traders, increasingly by credit, leaving traders with reduced cash flow and even financial losses from both unsold fish and extended credit (Fig. 1B4). Some mobile traders decided to shift to other occupations such as construction and fishing, likely to sustain their own household food security. Most mobile traders reduced the amount they worked from 6 or 7 days per week to 2 or 3, with some renting their motorbikes to others on off days (LK02). To match customer demand, they reduced the volume of fish they brought to resell, from 50–60 kg to 15–30 kg (Fig. 1B3). Some trader households began salting the leftover fish as a side business to sell to collectors (LK02-03, LK05-7). Though less common, traders with more capital set up new businesses, such as small restaurants or food stalls. As of July, some mobile traders had begun selling fruit to make up for lost fish profits or assisted with government food aid delivery for food aid as payment (LK02, LK03).

### Visayan Seas small-scale fisheries, Tañon Strait, Philippines

#### Pre-pandemic

The Philippines’ fishery sector, ranked 11th in total capture production globally (FAO 2020), is valued at USD 7.26 billion, and a relevant contributor to the national GDP (Lamarca 2017). Not only are the Philippines economically reliant on fish, they are ranked second globally for nutritional dependence on coastal and marine ecosystems (Selig et al. 2019).

In the Tañon Strait, an important Filipino fishing area, small-scale fishers catch several species, including groupers, mackerels, skipjacks, squid, sardines, tuna, rabbit fish, anchovy, and several other reef and pelagic fish (PH01, PH03). The fishers sell their catch through local consolidators to intermediaries in city markets, such as in Cebu City (PH01), normally fetching decent prices. These intermediaries require capital to purchase fish and transport it into city markets to supply restaurants, hotels, and retailers (PH01, PH05). Local consolidators may also provide loans or fishing gear to fishers to lock in low selling prices. Commercial fishers have a more formalized system, loading their catch into trucks at the port, or distributing to their regular market retailers. Canning factories own their vessels and directly transport catch from port to factory (PH01).

#### Since pandemic onset

In March, the Philippines’ government enacted COVID-19 mitigation measures for public health through an Enhanced Community Quarantine (ECQ).^2^ In addition to closing schools, non-essential businesses, and public transport across Luzon, curfews and travel restrictions were imposed, closing many provincial borders (Fig. 1C1). People were allowed out of their houses once a week for food and essentials, though in many parts of the country a pass was required. The army and police employed fines, arrests, and threats of violence to enforce the ECQ (Economist 2020). Concurrently, the country has faced its first economic slowdown in two decades (ibid) with approximately 16.6% of the population already living under the national poverty line (Asian Development Bank 2019). To partially mitigate these impacts, the government provided a subsidy/stimulus to poor families totaling USD 4.1 billion and implemented a food relief program for those in need, including wage earners (Fig. 1C6; Economist 2020).

Fishers were acknowledged as essential workers under the ECQ and exempted from stay at home orders through Resolution 15 of the government’s Inter-Agency Task Force on the pandemic (Fig. 1C2; Cabico 2020). The Bureau of Fisheries and Aquatic Resources (BFAR) issued permission to allow the movement of food and agri-fishery products (BFAR 2020a). However, implementation has been varied, with some fishers lacking access to resources or markets (Rey 2020; PH01, PH02, PH04). In one case, volunteers with a BFAR “Food Pass” were arrested for ECQ violation (BFAR 2020b). The SSF perception is that political favoritism is at play in receiving exemptions (PH01, PH02, PH03, PH04).

The government has adopted variably effective support mechanisms, including purchasing fish for food relief packages (Fig. 1C5), financial aid and loans, and new markets. Government-sponsored food relief packages have incorporated fish and promoted consumption of local fish (Department of Agriculture 2020), although implementation varied by locality. The Commission on Human Rights recommended that the national and local government units buy products from small-scale farmers and fishers for relief operations and mobile markets in rural and urban communities (Cabico 2020).

Fishers could receive around USD 100 in government aid through the Social Amelioration Program and were eligible for food aid. Small farmers and fisherfolk also had access to government loans of about USD 500 at zero interest, payable up to 10 years. The aid provided to families was barely enough to cover costs of a single person in a rural area, let alone in cities (PH01). While information about the loans was widely disseminated via radio, complying with requirements was difficult (PH01, PH03) and fishers in more remote areas were not reached (Fig. 1C6; PH01).

There have been several government-supported markets, including at the village level and mobile vendors, that have allowed fishers to redirect sales from their usual tourism and restaurant markets. The Department of the Interior and Local Government created a market for municipal fisherfolk and small farmers. BFAR Region 6 used an online system for consumers to pre-order and collect fish directly from their office (BFAR 2020c). In April, in Davao de Oro, BFAR Region 11, USAID and Silliman University started “Fish *Tiannge*,” an online marketplace connecting 6,000 fishers with buyers from 300,000 households in South Negros, Visayan Sea in the Visayas region, and the Calamianes Group of islands in Palawan (Fig. 1C5; Tamayo 2020). As a publicly accessible platform, customers purchase fish from sellers online and dedicated pedicab drivers deliver the orders. No prohibited fish products are sold and fish are sourced from locally licensed fishers. Fishing behavior has changed to adapt to shifting demand, yet what little is caught does not have a market. Consumers and intermediaries have either stopped buying completely (supported instead by aid) or are offering below market prices, citing transportation restrictions (Fig. 1C3). In Culion, fishers are struggling to sell their catch and fishing trips have decreased from 2–3 h to 30 min to reduce effort (Fig. 1C4; Rey 2020). In Cebu City, large fish prices dropped over 36% (PH03). Commercial operators with the resources to easily transport catch to market have stayed in business, whereas small-scale fishers, particularly informal (unregistered) fishers, are being questioned at the border by military or police (PH05). To bypass intermediaries, fishers turned to other non-governmental or community organizations. This effort has helped stabilize prices back at market value, but is limited by an organization’s resources (PH01, PH03).

### La Tortuga and La Islilla high seas artisanal fleet, Peru

#### Pre-pandemic

The Peruvian offshore artisanal fishery is the most profitable SSF in the nation, generating on average USD 452 million in exports annually from 2012 to 2016 (REDES 2018). Jumbo flying squid and pelagic shark are caught year-round with mahi caught seasonally (ibid). Prior to landing, boat owners pre-arrange sale to intermediaries who transport export-quality product to processing plants and lower quality to wholesale markets. Squid is primarily exported, with about 80% going to China, South Korea, and Spain in 2017 (PRODUCE 2018). Squid is used domestically as a filler in popular seafood dishes like *ceviche* or *arroz con mariscos*, while the lowest quality is used as a protein filler in fish meal (Grillo et al. 2018). In contrast, a larger portion (between 50 and 60%) of mahi flows into the domestic market; however, it has a greater chance of being mislabeled as higher-valued whitefish than exported mahi due to local preferences and perceived health risks (Marín et al. 2018).

#### Since pandemic onset

On March 16th, the President of Peru declared a state of national emergency, mandating cessation of all international travel, a national curfew, restriction of within-country travel except essential services, required use of personal protective equipment (PPE), and a stay-at-home order, dependent on relevant ministry recommendations (Fig. 1D1; Lerner 2020). Prior to the mandatory quarantine, the offshore artisanal fleet of La Islilla and La Tortuga had been operating normally, albeit with low landing volumes. Fishers clustered at fishing grounds and nearby landing sites in southern Peru fishing squid as the mahi season closed (PE02, PE03). Despite market closures in China and Europe, plants and exporters had continued to purchase mahi and squid, intending to continue exporting (PE01).

After the quarantine declaration, overall fishing effort in Peru dropped approximately 80% (Aroni 2020). Informants suggested three key reasons why this occurred for the offshore artisanal fleet: constricted market demand, confusion around best practices to prevent the spread of COVID-19, and the arrival of COVID-19 in vulnerable fishing communities (Fig. 1D2).

Fishing and product transport activities were considered essential services; however, conflicting means of emergency rule enforcement across scales of government led to confusion and breakdown of supply chain activities (Pino Shibata 2020; Riveros 2020; PE03, PE05). For example, to access transit permission from the Peruvian National Police, actors had to supply a personal national identification number, a tax code registry number, and a legal residence via online forms. As a largely informal sector, many actors were unable to provide this information (Gonzales 2020; Riveros 2020). Transit documentation allowed only for movement between a home and a single workplace, thus limiting shipping activities operating through two or more locations outside the worker’s legal residence (PE04, PE01). Movement restrictions and confusion amongst supply chain actors eventually led to stoppage of product movement and associated impacts on SSF livelihoods (Carrere 2020; Gonzales 2020).

Market constriction was experienced differently at supply chain nodes. Fishers reported reduced prices for squid and reduced demand across species (PE02, PE03, PE04). Some fishers attempted direct sale into local markets but attributed their limited success to lack of demand. Formal intermediaries who historically delivered product to processors were unable to work initially due to transit restrictions and lack of COVID-19 control protocols (Gonzales 2020). Other intermediaries were either unable to transport product to local open air markets or believed domestic consumers did not want squid (Carrere 2020; PE05). After 2 months, processors developed protocols and secured workforces allowing some plants to reopen at 70% capacity (PE01), thereby reactivating the squid supply chain and providing intermediaries a potential market. Although the government allowed international shipping to continue, processors and exporters reported a reduced international demand and warehoused product until their key international markets recovered. Local surplus kept squid prices low (PE01).

While plants and intermediaries developed internal practices to control the spread of COVID-19, fishers and dock workers relied on the National Agency of Seafood Sanitation (SANIPES). Protocol uncertainties led La Tortuga and La Islilla to take contrasting measures (Fig. 1D5); La Tortuga kept the community open, but enforced masks and social distancing, while La Islilla restricted all travel (PE03, PE4). SANIPES released national recommendations in April, however, within a month La Tortuga had over 14 fatalities attributed to COVID-19 and, a week later, the first cases appeared in La Islilla (PE06). These unfortunate losses increased local perception of health risks and disincentivized a return to offshore fishing (Fig. 1D3), despite aid (water, food, and PPE) being provided by nongovernmental actors and industry through corporate social responsibility programs (Fig. 1D4; PE03).

Despite the government-mandated quarantine and travel restrictions being lifted in July, the offshore artisanal fleet effort has not recovered (Gonzales 2020; Riveros 2020). Informants reported electing to stay at home and fish nearshore for subsistence, despite not receiving income or government loans/aid, because it was safer than potential exposure during long trips and traveling between multiple ports (Riveros 2020; PE02, PE04). Additionally, many struggled to finance the expensive trips, due to financial losses (Carrere 2020). Continued decreases in plant processing capacity and international demand for squid and mahi suggest that the impacts will continue (Fig. 1D6).

### Skipper Otto’s, British Columbia, Canada

#### Pre-pandemic

In Canada, British Columbia’s (B.C.) seafood industry relies heavily on export revenue. Seafood worth USD 1.1 billion was exported in 2018, with a third going to China, Japan, and Hong Kong (B.C. Ministry of Agriculture 2019). The viability of independent small-scale fisheries in B.C. has been threatened in recent decades due to inequitable licensing policies and increased control exerted by large corporations and seafood processors (Haas et al. 2016; Edwards and Pinkerton 2019; Standing Committee on Fisheries and Oceans 2019). Alternative seafood marketing initiatives, such as community supported fisheries (CSF), are a way to resist neoliberal fisheries structures by promoting non-market values within simplified value chains (Witter and Stoll 2017). Skipper Otto’s Community Supported Fishery (SO) is one such organization that has attempted to restructure the seafood supply chain and associated financial model by supporting SSFs through direct fisher-to-member sales.

SO sells seafood produced by nearly 20 B.C. fishing families to individual members from cities and small towns across several Canadian provinces. SO members purchase a “catch share” to order a variety of seafood over the course of a year. Membership fees and catch shares collected by SO at the start of the fishing season are used to help fishing families purchase their licenses and quotas, repair gear, and prepare for the upcoming fishing season. Fishers agree to sell a predetermined volume of their catch to SO, usually above expected market rates. SO collects seafood from its fishers/growers, sends it to public and private processing plants or processes it (filleting, vacuum sealing, flash freezing, etc.) through their own small-scale independent processor, and then stores frozen seafood at storage facilities or on site. Sales are directly made to members from the Granville Island Fishermen’s Wharf or through food co-ops and socially conscious businesses in Vancouver and domestically. Product labelling informs members who caught their seafood when, where, and with what gear. Such efforts are strategies to strengthen non-market values between fishers and seafood consumers within a seafood supply chain.

#### Since pandemic onset

COVID-19 reduced demand for niche B.C. seafood products like Dungeness crab, geoduck, spot prawns, and herring roe that would normally have been exported to Asian markets for Lunar New Year or sold locally to high-end restaurants (Fig. 1E1; Penner 2020). Fisheries, falling under the food sector, were declared an essential service and permitted to continue functioning (Government of Canada 2020). Additionally, the Canadian government released a USD 360 million aid package in mid-May to support Canadian fish harvesters and USD 80 million to assist the seafood sector in ensuring seafood supply, health and safety requirements of workers, market responsiveness, and domestic and international seafood storage capacities (Fig. 1E6; Prime Minister of Canada 2020). COVID-19 has minimally disrupted SO’s production and distribution systems. In the initial months of the pandemic, SO staff were aware of potential impacts COVID-19 could have on seafood systems and communicated the impacts and potential solutions to their members through blog posts and newsletters (Strobel 2020).

Since the pandemic and associated restrictions were introduced prior to the 2020 fishing season (April through October for several species), SSFs had time to adapt to the new conditions. Fishers supplying seafood to SO continued operating and received above-market prices for their catch (Fig. 1E2). Some fisheries, like spot prawn, delayed opening (from May to June 4) and fishers experienced considerably lower demand from export markets compared to previous years. To assist SO’s fisher partners and increase their own inventory, SO bought a larger portion of spot prawn fishers’ product to sell to members. With transportation and seafood processing also listed as essential services, SO’s local and regional seafood processing and distributions systems continued functioning (Fig. 1E4). SO introduced measures ensuring affordable access to seafood during the pandemic by extending their discounted early-bird membership sign-ups and providing less expensive, under-utilized seafood like hake and pink salmon.

SO also attempted to market and locally process niche seafood like Dungeness crab (SO01). With certain community pick-up locations being closed at the start of the pandemic, SO trialed a home delivery service to members in areas of Vancouver and partnered with new community pick-up locations. Toward the end of the summer, with a surge in new members (Fig. 1E3), a functioning home-delivery system, and more operational community pick-up locations, SO packing and distribution staff began working longer and more frequent shifts throughout the week, while maintaining social distancing measures (SO02). Increased seafood inventory and demand led to the installation of a second walk-in freezer at SO’s facilities (SO01). Based on availability, fresh seafood pick-ups occurred throughout the summer months, with longer operating hours, clear signage, and staff donning face masks and shields (pers. obs., S. Advani).

### Red sea urchin dive fishery, California, U.S.

#### Pre-pandemic

The United States (U.S.) is the top seafood importer and amongst the top five exporters worldwide (FAO 2018). California is not a top seafood-producing state, but one of the top eight employers in seafood processing and wholesale plants (NMFS 2018). The California red sea urchin (RSU) fishery operates in nine reporting regions across the state and, historically, has been one of the state’s most economically valuable fisheries valued at USD 14 million in 2001 (CDFW 2019). RSU total catch and value has since declined, averaging around USD 7 million annually between 2003 and 2017, with 2019 the worst year on record at USD 5 million (ibid). In 2016–2017, divers and processors in the northern reporting regions experienced drastic reductions in profits and a federal disaster was declared. Northern RSU actors qualified for federal relief, receiving USD 3.3 million (CDFW 2020a; pers. obs., H. Bassett; CA02), and the fishery is expected to qualify for retroactive federal relief statewide in 2018–2019 (pers. obs., H. Bassett).

The RSU fishery began in the 1970s to supply the Japanese market with urchin roe, or *uni.* Since the collapse of the Japanese market in the 1990s and concurrent burgeoning of sushi restaurants in the U.S., 90–95% of sales are to the domestic market, primarily to restaurants (CA01). Distributors maintain a small supply to Asian and SE Asian markets (ibid), and recently, local and direct-to-consumer operations have emerged. Run by divers or intermediary handlers (e.g., Sea Stephanie Fish^3^ or Tuna Harbor Dockside Market), they accounted for a relatively small market share prior to COVID-19 (pers. obs., H. Bassett).

Currently, there are 262 licensed divers (CDFW 2020b), with around 80 active in the fishery (CA02, CA03). Divers harvest *uni* operating either solo, with the assistance of a deckhand/tender, or in a small crew (CA02, CA03). Whole urchins are transported to processors, then shipped to distributors in the U.S., Asia, and Southeast Asia (CA01). Divers, processors, or intermediary handlers sell a smaller portion of the harvest as whole urchin to local restaurants or consumers. Some processors handle only *uni*, while at least one also processes and distributes many locally caught species. Divers tend to operate in more than one fishery or hold other part-time jobs in addition to diving for *uni* (pers. obs., H. Bassett; CA02).

#### Since pandemic onset

At the onset of COVID-19, the RSU fishery lost access to its primary and secondary markets: domestic restaurants and exports (Fig. 1F1). In mid-March, directed by state health and emergency officials, restaurants offered only drive-through or pick-up/delivery (CDPH 2020) and the governor issued a stay-at-home order (Executive Department State of California 2020), halting fishing and processing. Export became infeasible as stay-at-home mandates reduced international passenger flights, tripling the cost per pound for air freight across the Pacific (Bradsher and Swanson 2020).

With domestic restaurant and international distribution channels closed, only a small number of divers continued to harvest urchin and sell directly to consumers through personal connections, the Tuna Harbor Dockside Market, or intermediary fishmongers. The Market saw an increase in social media activity and a shift in their clientele as local San Diegans visited and tried to prepare *uni* for the first time (CA03). Capitalizing on the new attention, the Market offered pre-ordering online and curbside pick-up (ibid; Fig. 1F4). Sea Stephanie Fish shifted her sales toward consumers preparing *uni* at home and started an online store (Margolis 2020).

Some divers and processors pursued financial relief aid through Federal or State programs. In addition to enacting policies to mitigate the financial and health impacts of COVID-19 on individuals and families, California expanded unemployment benefits with the addition of USD 600 on top of the weekly benefit received by workers as part of the new Pandemic Additional Compensation initiated by the Federal CARES Act (Office of Governor 2020b). The State also provided USD 50 million in loan guarantees for small businesses not eligible for federal relief (Office of Governor 2020a). As independent operators, some divers were able to receive unemployment benefits and make more than they did diving, though it is unclear how many accessed benefits (CA02). Processors accessed small-business grants and loans to maintain operations through the CARES Act Paycheck Protection Program and Economic Injury Disaster Loans (CA01). One *uni-*specific processor closed for 1 month, but maintained their staff using aid funds, while another closed for 3 months and laid off most of their employees (CA01). One multi-species processor was able to maintain operations by adjusting to market demands (CA01). The California Sea Urchin Commission (CSUC), a statewide organization of divers, processors, and managers, provided information on requesting aid and it appears that access has not been an issue (CA01; CA02).

Domestic and international distribution remained limited, forcing processors and divers to adjust operations. Prior to COVID-19, divers fished on their own schedule and called the processor to let them know when to pick-up at the dock. Now they coordinate sales in smaller batches (Fig. 1F2; CA01, CA02). Fewer divers are working the RSU fishery overall and many continue to rely on other jobs or fisheries (though these are also impacted by COVID-19) and government assistance (Fig. 1F3; CA01, CA02, CA03). Some processors have started to add home deliveries in areas they delivered to restaurants (CA01). Social media has also facilitated direct sales and new customers (CA01; CA03). Direct impacts of COVID-19 on individuals are not thought to have been substantial, though one buyer in New York contracted the virus (CA01) and there are some signs of divers and individual actors experiencing increased stress and reduced mental health as a result of reduced stability and general anxiety (CA02).

## Discussion

Each of the SSF supply chains examined has been exposed to COVID-19 disruptions, across all supply chain nodes: directly disrupted by workers contracting the virus (PE), and indirectly by restrictions on consumers’ ability to purchase seafood, leading to decreased demand, and restrictions on mobility and trade, constricting need for production activities. Direct impacts were reduced in Peru through already distanced activities (e.g., fishing alone) or strong sanitary practices (e.g., wearing full PPE at processing plants), and existing cultural norms or health and sanitation policies helped in Canada, the U.S., and Indonesia. Sadly, the Peruvian case study saw several deaths in the fishing community, leading to fear amongst workers of perceived health risks.

Teleconnected vulnerabilities were apparent as some local supply chain actors experienced disturbances due to international market stoppages and restaurant closures. Impacts traveled through and between supply chains, exhibiting the broad reach of ripple effects in highly interconnected food distributions systems (Ivanov et al. 2014). While these patterns persisted across cases, vulnerabilities of actor groups differed due to varying factors. Long, complex, and opaque supply chains have made it difficult to determine further afield root causes of disruptions in most cases (e.g., Gephart et al. 2019; Triggs and Kharas 2020). Factors observed to increase or decrease supply chains’ and actors’ vulnerability are listed in Table 3.

**Table 3:** Factors contributing to an increase and decrease in exposure, sensitivity, and adaptive capacity of supply chain systems and actors observed across case studies. Supply chains in which a factor was observed are indicated by their abbreviations (see Table 1) in brackets following each factor. Due to differential vulnerability across actors within a system, in some cases, opposing factors have been observed within a supply chain

Exposure	
Factors contributing to an ***increase:***	Factors contributing to a ***decrease:***
• Reduced consumer financial wellbeing (indirect exposure) [LK, PE, PH] • Target international markets or restaurants (indirect exposure) [PE, LKR, PH] • Unsafe working or living conditions for supply chain actors (e.g., insufficient PPE or inability to distance from others while working) (direct exposure) [PE]	• Relatively high consumer financial wellbeing (indirect exposure) [SO, RSU] • Target local and domestic markets (indirect exposure) [all] • Sufficient PPE and safe working areas (e.g., ability to distance) (direct exposure) [RSU, SO]
Sensitivity	
Factors contributing to an ***increase:***	Factors contributing to a ***decrease:***
• Reliance on international markets and restaurants, especially tourism, which combines both [RSU, LKR, PE, PH] • Low income domestic and local consumer bases [LK, PE, PH] • Reliance on single species, especially niche or substitutable products [RSU, PE, LKR]	• Established domestic and local distribution strategies (largely direct to consumers) [SO, RSU, PH] • Diverse portfolios (fishers, processors, traders) [LKM, RSU, SO] • Government support to domestic and local supply chain actors and consumers [all] • Diverse distribution strategies [SO, RSU]
Adaptive capacity	
Factors contributing to an ***increase:***	Factors contributing to a ***decrease:***
• Established communication channels between intermediaries and producers to avoid supply/demand mismatches (organization, learning, flexibility) [RSU, LKM, SO] • Access to local and domestic consumers (organization) [PH, LKM, RSU, SO] • Government support to domestic and local supply chain actors (food—health; financial aid—assets; policies—agency, flexibility) [all] • Diverse distribution strategies (flexibility) [SO] • Diverse portfolios and livelihoods (flexibility) [RSU, LKM, SO] • Digital tools (learning, organization, flexibility) [RSU, PH, SO] • Storage and delivery capacity (assets) [SO, RSU] • Access to capital (assets) [SO] • Non-governmental, private industry, or community organization support or advocacy [PH, RSU, PE]	• Lack of access to local and domestic consumers (organization, learning, flexibility) [RSU, PE, LKR] • Restrictions on movement (agency, flexibility) [PE, LK] • Ineffective allocation of exemptions on movement restrictions for essential workers (organization, agency, flexibility) [PE, PH] • Lack of storage (assets) [PE, RSU, LKM, PH] • Lack of capital (assets) [PE, RSU, PH, LKM]

Using the theoretical framework (Table 2) to guide our analysis, four dimensions of SSF supply chains emerged as useful for discussing the key findings — livelihoods, aid and capital transfer, information and communication, and markets and distribution strategies, with diversification a key element throughout. We present the analysis according to these dimensions, and then summarize resilience and vulnerability implications.

### Livelihoods

Changes to livelihoods have been at the center of the COVID-19 crisis — they have been both the cause and result of disruptions to SSF supply chains, and for some, a means of adapting. The concept of a “livelihood” “seeks to bring together the critical factors that affect the vulnerability or strength of individual or family survival strategies… thought to comprise, chiefly, the assets possessed by people, the activities in which they engage in order to generate an adequate standard of living and to satisfy other goals such as risk reduction, and the factors that facilitate or inhibit different people from gaining access to assets and activities” (Allison and Ellis 2001). As the pandemic has strained livelihoods of people around the world, reducing their access to assets and activities, SSF supply chain actors’ livelihoods have been subsequently constrained, necessitating adaptive strategies to maintain their ability to generate an adequate standard of living.

Actors with diversified livelihoods have shown reduced vulnerability through decreased reliance on a single revenue stream (RSU), fishery (SO, RSU), or product (LKM, RSU). Fishing in multiple fisheries (RSU), processing multiple species (SO, RSU), and employing multiple distribution strategies (SO, RSU) have reduced actor sensitivity to market disruptions and increased their capacity to adapt. Actors involved with multiple SSFs and supply chains (LKM, SO, RSU) have shifted their efforts to nimbly follow markets as COVID-19 disruptions shift spatially and temporally. These findings align with other studies across numerous fields that have highlighted the positive impact of the “portfolio effect” on resilience in social-ecological systems that can experience sudden environmental and market shifts (e.g., Cline et al. 2017).

Many supply chains have attempted to respond to drops in demand by reducing their rate of usual supply chain activities. In several cases, actors have reduced the volume of fish moved through the chain, by reducing the time and effort spent in production and distribution (sometimes redirecting to other work; PH, PE, LKM, RSU). In the Philippines, self-owned and small businesses reduced purchases, while fishers supplying to tourist-reliant businesses and restaurants reduced their fishing effort. Langkat mobile traders also reduced the frequency of days worked and the amount of fish they bought to sell on their routes, and RSU divers fished only when they had pre-arranged a buyer. Largely, however, upstream-initiated reductions in catch production and sales have been unable to compensate for the reduced demand, with prices significantly decreasing in several cases (LKM, PH, and PE). This pattern suggests that COVID-19 has presented such a substantial shock that SSF supply chains and actors cannot maintain their core functions by simply coping and must take adaptive actions.

Some actors remaining within their usual supply chains have adapted by adjusting or diversifying their livelihood activities. The most common trend has been for actors to shift to new local distribution strategies, particularly direct-to-consumer sales. Others who already provided direct-to consumer sales have added new means of purchasing (e.g., online sales, public markets) and receiving (e.g., mobile delivery, curb-side pick-up) products. Some have expanded their offerings (e.g., salted fish in Langkat), provided tutorials for how to cook unfamiliar species (e.g., preparing *uni* at home in the RSU fishery), and increased their social media and customer outreach. These adaptive activities are actively shifting their SSF supply chain systems into new regimes.

Supply chain actors have also adapted by shifting to alternative job opportunities, repurposing their assets, or supporting their livelihoods with subsistence fishing. In Langkat, some mobile traders switched to fishing, while others transitioned out of the fishery sector completely, into construction, setting up small restaurants, or assisting with government food aid distribution. In Peru, offshore fisheries pivoted to nearshore artisanal raft fishing. In this way, artisanal fisheries have provided an essential service during the pandemic, supplementing livelihoods as needed, providing community food security (Steenbergen et al. 2019), and acting as a “labor buffer,” as they have during previous shocks (Béné et al. 2016). Making these livelihood shifts requires not only awareness of and access to other livelihood options, but the capacity to adapt to their new requirements.

A multi-pronged macroeconomic shock like COVID-19 highlights how crucial diversification is for resilience. For example, the ability for supply chain actors to move to other lines of work (i.e., diverse livelihood options) may initially seem to not support the continued operation of the supply chain. However, the ability of the actor to be flexible and support their livelihood outside of the fishery when demand is low supports the near-term wellbeing of that actor, and their ability to return in accordance with demand, thereby supporting supply chain resilience (e.g., Cinner and Barnes 2019). In contrast, when individual actors or supply chain nodes fail, supply chain resilience is reduced (e.g., Golan et al. 2020).

### Aid and capital transfer

Targeted food aid (Global Panel 2020), direct fiscal aid, and supportive policies (Triggs and Kharas 2020) proved essential in supporting adaptation of SSF supply chains and actors to the widespread and varied disruptions of COVID-19. Aid has been supplied across all cases as financial support, food distribution, technical advice, or supportive policies, and has been provided by governments, NGOs, international institutions, fishery cooperatives, private enterprise, family members, and others. National programs distributed COVID-19-relief aid to supply chain actors and the community at large. While aid has been critical, impacts have varied widely based on the type of aid provided, the efficacy and equity of distribution, and the larger economic and social context.

Financial aid to intermediary small and medium businesses, via grants, loans, and direct cash transfers, was a critical factor in continued operations, reducing both exposure and sensitivity to the closure of international markets and reduced demand. In the U.S., unemployment aid and small business loans has been directed to fishers and processors. The Canadian Seafood Stabilization Fund of nearly USD 80 million was offered to seafood processors. This package was in addition to a USD 360 million COVID-19 fund to support Canadian fish harvesters. In Peru and the Philippines, financial aid was provided by the government although receipt was extremely limited, with plants primarily receiving aid, versus fishers. Access to subsidies was undercut by informality in the sector with unregistered actors excluded.

The effectiveness of government (or other) aid in reducing supply chain and actor vulnerability was limited by constraints on aid recipients and scale mismatches with COVID-19 shocks; aid has been supplied to some, though not all, domestic recipients, however cannot reach international markets where many of the disruptions are rooted. Without substantial inter-governmental coordination, national governments and organizations have limited ability to mitigate for negative impacts of a supply chain’s reliance on collapsed international markets (Triggs and Kharas 2020), thus the increased risk posed to local communities by teleconnected vulnerabilities (Adger 2006).

Given the limits on aid in combatting international market losses, financial aid coupled with consumer financial security may have been an underlying factor in supply chains demonstrating relatively less sensitivity to COVID-19. Supply chains located in high-income countries (HICs) with strong unemployment aid and disaster relief infrastructure (e.g., SO and RSU) had access to consumers who retained more purchasing power than those in the low- and middle-income countries (LMICs) of Indonesia, the Philippines, or Peru. The U.S. and the Philippines provided only one-time stimulus payments, unlikely to change buying power in an ongoing crisis (Triggs and Kharas 2020). In the LMICs, reduced income and purchasing power amongst consumers both reduced their access to food and indirectly impacted supply chain actors reliant on related markets (Béné 2020). In the Philippines, government and NGO financial investments supported new online markets and one fisher organization subsidized the price of fish, offsetting fisher losses from reduced market prices. Only in the SO case were local demand, consumer purchasing power, and access to consumers sufficient to maintain a high volume of sales and support operations without subsidization or reduced incomes. Direct-to-consumer sellers in the RSU fishery saw similar success, though processors and handlers without established connections with local consumers were less able to capitalize on the potential consumer base.

In addition to fiscal aid, household food aid was distributed and supportive SSF supply chain policies were passed in Indonesia, Peru, and the Philippines. The provisions were intended to reduce the vulnerability of recipients, but in some cases, unintended ripple effects were evident. In the Philippines, several local government units purchased local fish to distribute as part of the food aid, although at reduced prices. Including seafood in food relief protected access to nutritious foods for recipients, as recommended by the Global Panel (2020), and simultaneously supported local fisheries. In contrast, food aid in Langkat included tofu, tempeh, and eggs, which supported consumers’ and supply chain actors’ nutritional needs, but not SSF livelihoods. The government’s provision of alternative protein may also have indirectly led to further reduced fish purchases via reduced demand. As with fiscal aid, food aid had limited reach to informal actors or those in rural areas, requiring paperwork and registration. Similarly, movement restriction exemptions allowed many to continue working, but distribution of benefits was varied and inequitable. In some cases (e.g., the Philippines and Peru), bureaucratic systems and sector informality made exemptions inaccessible.

Government aid has been the primary source of system-level support for maintaining supply chains in the face of the COVID-19 crisis, highlighting both the crucial role of relief aid and its limitations. Governments and local authorities have largely striven to protect the SSF sector (Béné 2020), with most problems arising from issues with implementation, aid strategies, or existing systemic issues. Having existing infrastructure to distribute aid seemed to benefit associated supply chain actors — thereby bolstering system resilience and reducing vulnerability — while the informality of some SSF supply chains seemed to inhibit effective government support. Limitations in immediate, equitable, and coordinated government response, as recommended by Adger (2006) and Triggs and Kharas (2020), resulted in non-governmental organizations stepping in to support actors (RSU, PH, PE). In Peru, private entities (plants) provided technical assistance for safety protocols and coordinated PPE distribution and, in the RSU fishery, the CSUC shared information for accessing unemployment aid. Effective distribution of appropriate and ongoing aid and supportive policies, whether governmental or otherwise, emerged as an important factor in reducing systems’ and actors’ sensitivity to disruptions and bolstering their adaptive capacity.

### Information and communication

Access to information (learning), knowledge sharing (knowledge), and coordination between actors (organization) have been essential dimensions of adaptive capacity employed to manage uncertainties around potential COVID-19 mitigation strategies and maintain supply chain function. Internet and communication technologies (ICTs) were central to the adaptive actions of supply chain actors. Digital tools are increasingly used for coordination and transformation within supply chains (Jensen 2007; Omar and Chhachhar 2012; Nthane et al. 2020), and in the presented cases, were used to communicate crucial information about supply and demand, share knowledge of potential COVID-19 policies and impacts, create new distribution strategies, and access new consumer bases. Government agencies provided COVID-19 mitigation information for SSFs across the cases, with ICTs essential in Peru and the Philippines as agencies used WhatsApp and Facebook to communicate to fishers. In the RSU, information was shared via pre-established e-mail listserv and public meetings were held via Zoom to discuss allocation of federal relief. ICTs were also commonly harnessed to bolster online sales and reach new clients through social media campaigns, drawing on organizational capacity to successfully expand engagement strategies and share information with consumers. Philippine SSFs leaned on the government and NGOs’ organizational capacity and assets to create online markets, maintaining product flow to local supply chains and organizing safe pick-up processes. Actors in the SO and RSU cases used pre-existing assets and digital networks to expand their consumer bases.

The use of available tools aided mitigation of potential inequities in access to information. If not considered, lack of local infrastructure, individual socioeconomic class, and historical power distributions can inhibit access to ICTs and uniform distribution of information (Nthane et al. 2020), creating disparities in adaptive capacity. However, in these cases information appears to have been largely accessible. In the Philippines, the government used radio programming to broadcast loan program details. In Indonesia, actors received information through community networks providing better access to ICT-sourced information, as has been seen in other studies (Nthane et al. 2020). In accessing knowledge and supporting their learning, actors relied on the assets and organizational capacity of their networks to bolster their individual adaptive capacity.

Limited supply chain coordination reduced actors’ capacity to adapt to product supply and demand disruptions. Perishable or live seafood products require special storage, and SSF supply chain nodes are highly sensitive to fluctuations in market demand. Adaptation via increasing storage capacity requires substantial assets and has only been seen in one case (SO). Otherwise, producer and buyer coordination is the primary means of ensuring harvest will find a market and garner profits. Supply chain actors who were able to coordinate the supply of fish to available buyers (e.g., RSU) were better able to navigate fluctuations in demand and reduce product waste and effort. Several SSFs saw initial seafood gluts when the markets dropped-off suddenly, with more complex supply chains and distanced nodes taking longer to minimize supply–demand mismatches (e.g., PE). Additionally, higher levels of adaptive capacity on the side of all supply chain actors (including consumers) seems to have improved coordination, as these actors were more able to communicate their preferences. For example, wealthier Indonesian clients preferring a higher value product communicated their demand to mobile traders, who did not typically carry them. Similarly, SO, relying on e-commerce and a cooperative business model, was able to effectively communicate with their suppliers to meet the demand of a growing client base and communicate surplus to clients to encourage sales.

### Markets and distribution strategies

Market diversification, in particular, has been highlighted as a key factor for SSF resilience to surprise market disruptions, both in our study and others on COVID-19 (e.g., Béné 2020; Knight et al. 2020). Within our case studies, actors have demonstrated reduced vulnerability to supply chain disruptions by their ability to adapt to alternative markets, innovate with new strategies to sell products, and utilize agency and assets to diversify the products they handle. Supply chains and their actors heavily reliant on international trade or travel were more sensitive to supply chain disruptions, as has been noted elsewhere (Stoll et al. 2018). In B.C., fishers sensitive to international market demand were unable or unwilling to sell their product internationally, but accessed new local and domestic markets through SO. SO arguably benefited from its low exposure to international market variations and could purchase larger volumes of seafood at lower prices to sell domestically. Langkat mobile traders continued tapping into local markets with a diverse set of products, while processors reliant on seafood exports were sensitive to trade disruptions. Peru’s squid and mahi supply chains and actors were additionally sensitive to trade and transit restrictions because of the centralization of processing facilities along the Peruvian coast. In the Philippines, fisheries dependent on tourism for sales redirected their catch to local communities, though often at significantly reduced prices. Similarly, the RSU fishery was highly sensitive to COVID-19 closures as restaurants are a majority of their market.

Actors in each case operationalized different available dimensions of adaptive capacity to diversify their distribution strategies and product portfolios, thereby reducing their vulnerability to seafood supply chain disruptions. As mentioned previously, social media and ICTs, like WhatsApp, were used to access new markets and facilitate purchases, as seen in the Philippines, SO, and RSU. Recognizing the limited mobility of consumers, SO relied on agency and assets to implement new targeted distribution strategies, starting home delivery service and adding customer pickup locations. In some cases, actors could change their species or products offered in response to shifting demand volume. Langkat mobile traders already sold an assortment of perishable goods, including multiple fish species, selecting the species and volume each morning depending on price, quality, and expected sales. As fish sales declined, they demonstrated increased flexibility and agency by adding fruit sales, with similarly high profit margins. Alternately Rio, the Indonesian crab and snail processor, lacked the flexibility to shift to new species or markets and closed his factory. In Peru, the supply chain had a limited, inflexible portfolio of two species of frozen seafood leading many plants to shut down or reduce capacity when demand disappeared. Peruvian squid and RSU, as single species fisheries, were also limited by their inflexibility to target different species. In Canada, SO started selling less expensive species, like hake, and is exploring options to begin custom processing Dungeness crab, previously sold unprocessed. As previously documented, SSFs can benefit from or be impacted by global seafood trade in varied ways (Crona et al., 2016a). In our cases, SSF supply chains had mixed responses to market and distribution disruptions, indicating that some chains may not be sufficiently prepared for the increased risk associated with exposure to international markets and teleconnected vulnerabilities.

### Resilience and vulnerability

All cases examined can be considered to have shifted to a “radically different state” in the wake of COVID-19 due to substantial changes in (1) the type or volume of product traded (all, with an increase seen only in SO), (2) the process by which trade occurs (all, except SO), and (3) the groups of actors involved (all, with an increase in participation only in SO). While the magnitude of disruption associated with COVID-19 has been too great for the SSF supply chains to absorb without necessitating adaptive action and movement into new regimes, their ability to continue performing core functions has varied as a result of disparate vulnerabilities:

Three cases (PE, LKR, and PH) exhibited relatively low resilience and high vulnerability in that supply chain actors could not continue working in their usual roles and the usual consumers did not receive their regular seafood products. All three of these cases were reliant on international markets or tourism (high exposure and sensitivity) and exist in relatively low-income countries and local areas (low adaptive capacity), where they have been unable to establish new markets either due to consumer preference, spending power, or both. With some established domestic market links, the Philippines case fared somewhat better than the other two (lower sensitivity and higher adaptive capacity).

Two cases (LKM and RSU) demonstrated moderate resilience and vulnerability as some actors continued in the supply chain and some consumers maintained access to products. Both cases target domestic and local markets primarily, but their usual distribution channels were disrupted by reduced consumer spending power (LKM) and restaurant closures (RSU) (moderate exposure and sensitivity). Adaptive capacity varied amongst actors and allowed some to remain active in the supply chain by modifying the focus of their activities, while others employed their agency and flexibility to pursue other livelihood activities or means of support. Diversification of products (LKM) and distribution strategies (RSU) was key to continued operation of the supply chains (moderate adaptive capacity).

One case (SO) showed high resilience and low vulnerability and was able to increase operations, hiring new employees and accessing new consumers. Targeting an affluent domestic market via established diverse distribution strategies and channels (low exposure and sensitivity, high adaptive capacity), the SO supply chain was able to capitalize on reduced international trade, increasing domestic market demand and reducing local catch price. Their market was further strengthened by ongoing government financial relief provided to Canadian citizens (lower sensitivity).

Examining across all case studies, several key factors for reducing SSF supply chain and actor vulnerability to the pandemic have emerged: (1) access to local and domestic markets with purchasing power (increased adaptive capacity, reduced sensitivity); (2) low reliance on international, tourism, and restaurant markets (reduced exposure and sensitivity); (3) access to information and communication (increased adaptive capacity); (4) government aid supporting the SSF supply chain directly or consumer spending (reduced sensitivity, increased adaptive capacity); and (5) diversified distribution channels and strategies, livelihood options, and products (increased adaptive capacity, reduced sensitivity).

## Conclusion

COVID-19 is an unprecedented supply chain disruptor, but will not be the last to transcend geographic boundaries. Climate change, slower moving in its effects, will have similar global reach (Lam et al. 2020). Similar processes as those observed here have been shown to be critical for building general resilience to climate change in marginal communities; namely, social learning, and communication across multiple institutional scales, community reorganization, and adaptive capacity (Osbahr et al. 2008).

The negative impacts experienced by SSF supply chain actors since the onset of COVID-19 have not been limited to direct health impacts. Instead, impacts derived mostly from underlying social conditions that increased the vulnerability of systems and actors to this type of disruption, as has been theorized by conceptual models (Blaikie et al. 1994; Béné 2020). These underlying factors contributed to increased or decreased vulnerability of actors and, ultimately, different levels of system resilience and vulnerability. SSF supply chains and actors have modified their activities and roles in response to COVID-19 disruptions, but whether these changes will be sustained going forward is unclear. Similarly, whether and how much any sustained changes will benefit different supply chain actors remains to be seen.

These case studies suggest several systemic changes that could support reduced vulnerability in SSF supply chains and prepare actors for future shocks. First, development of well-connected local and domestic supply chains reduces reliance on international markets and their associated teleconnected vulnerabilities. While local or domestic distribution may not be economically preferable or culturally viable during “normal” times, efforts to develop the market and diversify distribution strategies may provide valuable alternatives to the globalized trade system, leaving systems and actors less vulnerable to shocks and better able to cope or adapt in times of stress. Second, supporting diversified product portfolios, livelihood activities, and distribution strategies will increase actor and system flexibility and their agency to respond to market fluctuations and logistical limitations. Third, proactive establishment of effective means of equitably distributing aid and information will support both supply chain actors and consumers to continue to operate their respective roles, thereby decreasing supply chain disruption. Providing sufficient and consistent financial aid to households will increase their ability to purchase seafood and either buying fish for food relief packages or directly providing aid to supply chain actors will promote their continued functioning.

The case studies presented here suggest that these lessons for supporting invulnerable and resilient SSF supply chains can apply to a broad range of systems and contexts, if appropriately adapted. As is always the case with social-ecological systems, there is a positive feedback loop in which the wellbeing of system actors supports continued provision of wellbeing from the system in which they participate; thus, equity and inclusivity are essential considerations to promote sustainable and resilient systems. The pandemic has highlighted flaws in globalized and integrated seafood systems and its outcomes should inform preparation for similar future shocks.

## Data Availability

Interview data is not shared to maintain interviewee privacy and confidentiality.

## Footnotes

1 Names have been changed to maintain anonymity.

2 The Philippines COVID-19 measurements ranged from General Community Quarantine (GCQ) to the more restrictive Enhanced Community Quarantine (ECQ), with a Modified ECQ defined as a “new normal” restriction level.

3 https://www.seastephaniefish.com/
